# Myristoylation drives dimerization of matrix protein from mouse mammary tumor virus

**DOI:** 10.1186/s12977-015-0235-8

**Published:** 2016-01-05

**Authors:** Michal Doležal, Aleš Zábranský, Jiří Dostál, Ondřej Vaněk, Jiří Brynda, Martin Lepšík, Romana Hadravová, Iva Pichová

**Affiliations:** Institute of Organic Chemistry and Biochemistry, Academy of Sciences of the Czech Republic, v.v.i., Flemingovo nám. 2, 166 10 Prague, Czech Republic; Department of Biochemistry, Faculty of Science, Charles University in Prague, Hlavova 8, 128 40 Prague, Czech Republic

**Keywords:** Dimerization, Matrix protein, MMTV, Molecular dynamics, Mouse mammary tumor virus, Myristoylation, Myristoyl switch, Retrovirus, Analytical ultracentrifugation, X-ray crystallography

## Abstract

**Background:**

Myristoylation of the matrix (MA) domain mediates the transport and binding of Gag polyproteins to the plasma membrane (PM) and is required for the assembly of most retroviruses. In betaretroviruses, which assemble immature particles in the cytoplasm, myristoylation is dispensable for assembly but is crucial for particle transport to the PM. Oligomerization of HIV-1 MA stimulates the transition of the myristoyl group from a sequestered to an exposed conformation, which is more accessible for membrane binding. However, for other retroviruses, the effect of MA oligomerization on myristoyl group exposure has not been thoroughly investigated.

**Results:**

Here, we demonstrate that MA from the betaretrovirus mouse mammary tumor virus (MMTV) forms dimers in solution and that this process is stimulated by its myristoylation. The crystal structure of *N*-myristoylated MMTV MA, determined at 1.57 Å resolution, revealed that the myristoyl groups are buried in a hydrophobic pocket at the dimer interface and contribute to dimer formation. Interestingly, the myristoyl groups in the dimer are mutually swapped to achieve energetically stable binding, as documented by molecular dynamics modeling. Mutations within the myristoyl binding site resulted in reduced MA dimerization and extracellular particle release.

**Conclusions:**

Based on our experimental, structural, and computational data, we propose a model for dimerization of MMTV MA in which myristoyl groups stimulate the interaction between MA molecules. Moreover, dimer-forming MA molecules adopt a sequestered conformation with their myristoyl groups entirely buried within the interaction interface. Although this differs from the current model proposed for lentiviruses, in which oligomerization of MA triggers exposure of myristoyl group, it appears convenient for intracellular assembly, which involves no apparent membrane interaction and allows the myristoyl group to be sequestered during oligomerization.

**Electronic supplementary material:**

The online version of this article (doi:10.1186/s12977-015-0235-8) contains supplementary material, which is available to authorized users.

## Background

Mouse mammary tumor virus (MMTV), a causative agent of mammary breast cancer and T cell lymphomas in mice (for review see [1]), is a representative of the Betaretrovirus genus. In contrast to HIV-1 and other retroviruses that assemble immature particles during budding at the plasma membrane (PM), betaretroviruses assemble immature particles within the cytoplasm prior to transport to the PM for budding. In general, the hexameric lattice of immature retroviral particles is formed by association of 1500 to 2500 molecules of the multidomain structural polyprotein Gag. The N-terminal domain of Gag, matrix (MA) protein, is responsible for Gag targeting and PM binding. During or shortly after budding, the viral protease-dependent cleavage of Gag initiates rearrangement of individual mature proteins, after which MA molecules remain associated with the enveloping membrane and form the outer shell of the viral particle [2].

In most retroviruses, the N-terminal glycine residue of the MA domain of Gag is co-translationally modified with myristic acid (reviewed in [3]). Myristoylation of MA and the highly basic patch of amino acids located near its N-terminus mediate the interaction of Gag with the PM. The myristoyl group serves as a hydrophobic anchor associated with the lipidic acyl chains in the PM, while the positively charged surface patch in MA interacts electrostatically with the polar heads of phospholipids. Both the myristoyl group and the basic patch function in concert to facilitate efficient MA membrane anchoring (for review see [4]). In HIV-1 and other viruses that assemble particles at the PM, prevention of myristoylation by mutation of the N-terminal glycine to alanine (G2A) dramatically reduces binding of Gag to PM and inhibits viral particle formation [5–9]. However, in betaretroviruses such as Mason-Pfizer monkey virus (M-PMV) and MMTV, the analogous G2A mutation does not interfere with the process of intracytoplasmic assembly but completely blocks the transport of viral particles to the PM [10, 11]. The MA domain not only targets the PM and mediates the association of viral particles, but it also dictates the lipid-binding specificity (for review see [12, 13]).

In addition to its role in promoting the association with the PM, some other functions have been attributed to the MA domain of Gag. In M-PMV, a highly conserved short stretch of amino acids within MA domain, termed the cytoplasmic targeting/retention signal (CTRS), interacts with Tctex-1, a component of the dynein motor machinery [14], and is responsible for intracellular targeting of Gag to the pericentriolar region for assembly. Similarly, CTRS in the MMTV MA domain was recently found to mediate pericentriolar targeting of Gag [15]. Several mutations within the M-PMV MA domain can abrogate transport of assembled particles to the PM in a manner similar to the G2A mutation in Gag, and these changes in MA might negatively affect Gag/Env interaction at the recycling endosome and prohibit usage of the vesicular transport system [16–18]. The lentiviral MA domain may be directly involved in incorporation of Env into virions, at least for HIV-1 [19].

Structures of MA protein from ten retroviruses have been resolved by NMR or X-ray crystallography [20–29]. Despite the low sequence homology of these proteins, their overall three-dimensional organization is remarkably similar, consisting of a globular core composed of four or five α-helices (reviewed in [12]). However, structures of myristoylated forms [myr(+)] have been reported only for MA proteins from the lentiviruses HIV-1 [30], HIV-2 [28], feline immunodeficiency virus (FIV) [31], and the betaretrovirus M-PMV [32]. The solution structure of myr(+) HIV-1 MA revealed an equilibrium between a monomeric form with the myristoyl group sequestered inside the protein core and a trimeric form with exposed myristoyl groups [30]. Transition between the monomeric and trimeric states of myr(+) HIV-1 MA is entropically regulated, and higher protein concentration stimulated and stabilized formation of the trimeric form [30]. In contrast, nonmyristoylated [myr(−)] HIV-1 MA appeared mainly in the monomeric state in solution [20] but in the trimeric state in crystals [33]. Despite the structural similarity between the HIV-1 and HIV-2 MA proteins, myristoylation does not influence the trimerization ability of HIV-2 MA [28]. However, betaretroviral MA from M-PMV readily forms oligomers (dimers and trimers) in its myr(−) form [34], while myr(+) MA is monomeric in solution [32]. Based on extensive studies of HIV-1 MA, it is generally accepted that the myristoyl group can adopt a sequestered conformation within the globular head of MA as well as an exposed conformation where it is more accessible and can facilitate membrane association. The process of transition between these two states, termed the myristoyl switch, can be influenced by several factors, including protein concentration, oligomerization status, and pH [30, 35].

Here, we investigated the role of myristoylation on oligomerization of MMTV MA and determined the crystal structure of myr(+) MMTV MA, which represents the first X-ray structure of a myr(+) retroviral MA protein. Our structural data reveal the sequestered conformation of the myristoyl group bound into the MMTV MA dimer interface, and analytical ultracentrifugation and molecular dynamics analyses show its crucial role for MA dimerization. Based on the structural and molecular modeling data, we constructed MMTV MA mutant with a reduced ability to form dimers in vitro. Identical mutations were also tested in the context of MMTV virus and their impact upon assembly and transport of immature particles was analyzed.

## Results

### Myristoylation stimulates dimerization of MMTV MA in solution

**Fig. 1:**
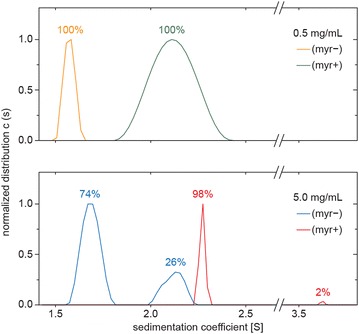
Dynamic equilibrium of myristoylated myr(+) and nonmyristoylated myr(−) MA sedimenting species analyzed by analytical ultracentrifugation. Normalized continuous size distributions (c(s)) resulting from sedimentation velocity analyses were compared for myr(−) and myr(+) MAs at concentrations of 0.5 and 5.0 mg/mL. The percentages of monomeric, dimeric, and higher forms for individual MA forms in a single experiment are indicated above the peaks

Typically, myristoyl group participates in protein subcellular localization by facilitating protein–membrane and protein–protein interactions (reviewed in [36]). To determine the influence of myristoylation on self-association of the MA domain of MMTV Gag, we performed analytical ultracentrifugation using bacterially expressed and purified myr(+) and myr(−) MA, containing five C-terminally attached amino acids from the pp21 domain followed by a His-tag [37]. The purified myr(+) MA contained less than 0.1 % of myr(−) MA as documented by mass spectrometry analysis (Additional file 1: Figure S1). Sedimentation velocity experiments were performed with recombinant myr(+) and myr(−) MA proteins at two different concentrations (0.5 and 5.0 mg/mL), and we analyzed their tendency to form oligomers by fitting the sedimentation data using a continuous size distribution model. The comparison of all resultant size distributions is shown in Fig. 1. At the lower concentration, myr(−) MA formed one discrete particle with sedimentation coefficient s_20,w_ = 1.57 S. This value corresponds well with the mass of the MA monomer, pointing to a moderately elongated particle with approximate dimensions of 2–3 × 4–6 nm. At the higher concentration, myr(−) MA formed two particles, corresponding to a monomer (s_20,w_ = 1.69 S; 74 %) and a dimer (s_20,w_ = 2.12 S; 26 %). In contrast, at the lower concentration, myr(+) MA formed only dimers with s_20,w_ = 2.12 S. At the higher concentration, myr(+) MA predominantly formed dimers (s_20,w_ = 2.27 S; 98 %) but also formed a particle corresponding to a tetramer (s_20,w_ = 3.69 S; 2 %). Thus, we conclude that the ability of MMTV MA to form dimers is stimulated by its myristoylation.

The dimerization of MMTV MA driven by its myristoylation also provides a likely explanation for the separation of His-tagged myr(+) and myr(−) MMTV MAs on an IMAC column that we described previously [37]. A dimer of MA has two His-tags which results in higher affinity to the IMAC column. The efficiency of the separation is influenced by the difference in the ability of myr(+) and myr(−) MAs to form dimers. The formation of myr(+):myr(−) mixed dimers decreases the separation efficiency.

### Structure of myristoylated MMTV MA

To provide structural evidence for the role of the myristoyl group in MA dimerization, we determined the structure of myr(+) MA prepared by the procedure described by Dolezal et al. [37]. The protein crystallized in the *P3*_*1*_*21* space group with two molecules in the asymmetric unit. The structure was solved by single isomorphous replacement with anomalous scattering (SIRAS) using potassium iodide. The final dataset was collected to 1.57 Å resolution, and the final model was refined to R = 22.6 % (R_free_ = 26.3 %). Crystal parameters, data collection statistics, and refinement statistics are summarized in Table 1. In the refined structure, myristoyl groups and their connections to the N-terminal glycines were well-defined in the electron density map, as were the first 91 of 111 amino acid residues. The last 20 residues (the C-terminus of the protein and the His-tag) lacked electron density, likely due to a disordered conformation. The structure was deposited in the Protein Data Bank under PDB ID 4ZV5.

**Table 1:** Crystal parameters, data collection statistics, and refinement statistics

Crystal	Native	I^−^ soaked	Native
Data collection statistics			
Space group	*P3*_*1*_ *21*	*P3*_*1*_ *21*	*P3*_*1*_ *21*
Cell parameters (Å, °)	61.90 61.90 89.89 90.0 90.0 120.0	61.88 61.88 89.84 90.0 90.0 120.0	61.92 61.92 90.05 90.0 90.0 120.0
Wavelength (Å)	1.5000	1.5000	0.9184
Resolution (Å)	46.04–1.90 (2.01–1.90)	46.02–1.86 (1.97–1.86)	46.08–1.57 (1.66–1.57)
Number of unique reflections	15674 (2178)	17201 (2727)	28486 (4526)
Multiplicity	5.7 (5.2)	4.9 (3.9)	6.2 (5.7)
Completeness (%)	98.4 (84.3)	99.7 (99.2)	99.7 (99.1)
R_merge_^a^	3.6 (20.1)	6.0 (32.8)	3.9 (66.7)
Average *I*/σ(*I*)	29.9 (7.7)	15.9 (3.4)	23.9 (2.4)
Wilson B (Å^2^)	30.6	35.5	30.3
Refinement statistics			
Resolution range (Å)	34.44–1.90 (1.95–1.90)		46.08–1.57 (1.61–1.57)
No. of reflections in working set	14,890 (696)		25616 (1826)
No. of reflections in test set	748 (36)		1423 (92)
R value (%)^b^	26.0 (60.7)		22.6 (29.4)
R_free_ value (%)^c^	32.4 (70.7)		26.3 (33.4)
RMSD bond length (Å)	0.024		0.012
RMSD angle (º)	2.66		1.6
Number of atoms in AU	1534		1634
Number of protein atoms in AU	1494		1512
Number of water molecules in AU	40		94
Mean B value (Å^2^)	23.4		24.2
Ramachandran plot statistics^d^			
Residues in favored regions (%)	96.7		99.4
Residues in allowed regions (%)	1.6		0.0
PDB ID			4ZV5

The data in parentheses refer to the highest-resolution shell

^a^R_merge_ = $\sum\nolimits_{\text{hkl}} \sum\nolimits_{\text{i}} I_{\text{i}} \left( {\text{hkl}} \right) \, - \langle {I\left( {\text{hkl}} \right) \rangle |/\sum\nolimits_{\text{hkl}} \sum\nolimits_{\text{i}} I_{\text{i}} \left( {\text{hkl}} \right)}$, where $I_{\text{i}} \left( {\text{hkl}} \right)$ is the individual intensity of the ith observation of reflection hkl and $\left\langle {I_{\text{i}} \left( {\text{hkl}} \right)} \right\rangle$ is the average intensity of reflection hkl with summation over all data

^b^R-value = ||*F*_o_| − |*F*_c_||/|*F*_o_|, where *F*_o_ and *F*_c_ are the observed and calculated structure factors, respectively

^c^R_free_ is equivalent to R-value but is calculated for 5 % of the reflections chosen at random and omitted from the refinement process

^d^Determined by MolProbity

**Fig. 2:**
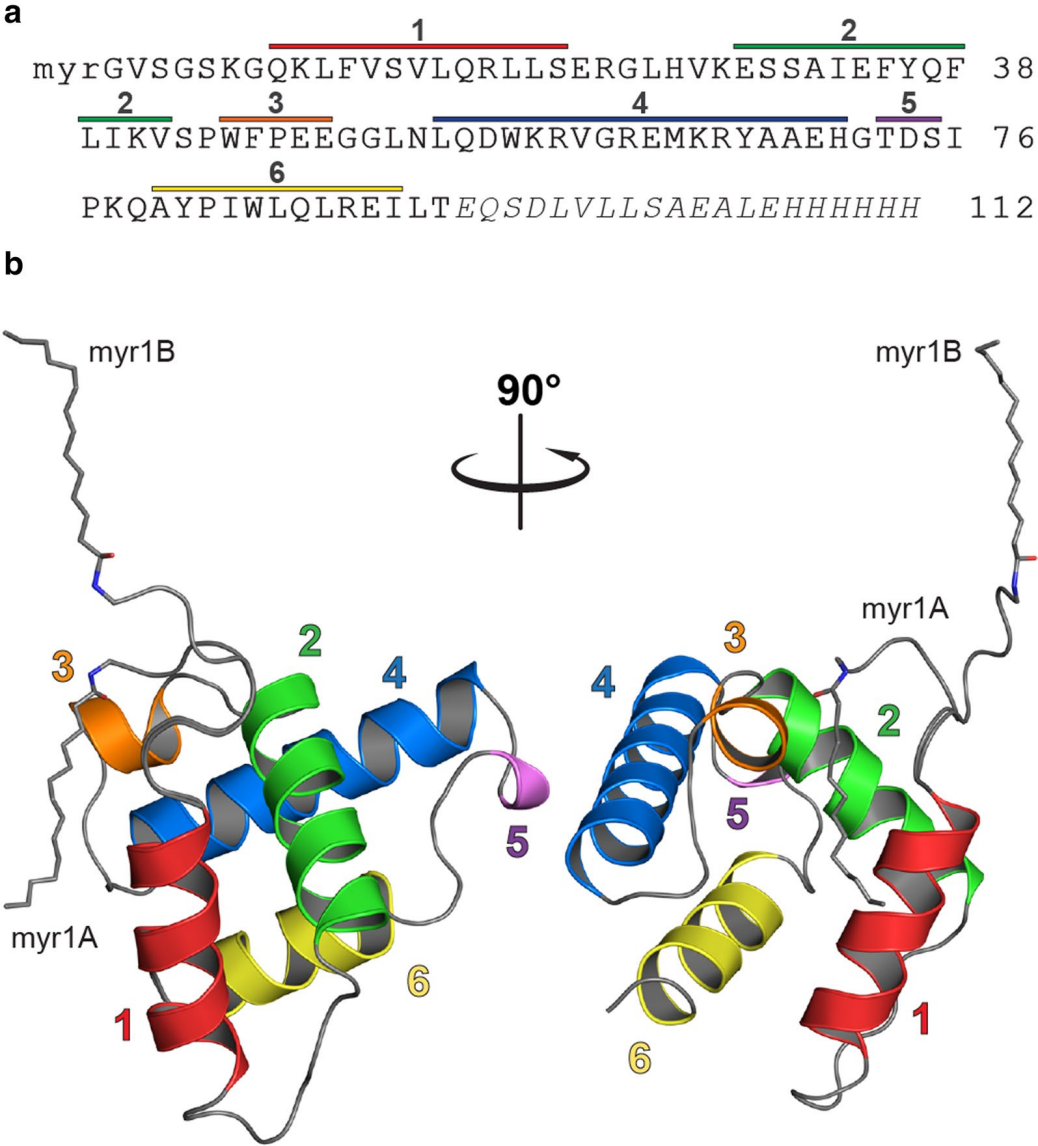
Structure of myristoylated MMTV MA. **a** The amino acid sequence of MMTV MA with a C-terminal His-tag. The N-terminal glycine is designated as residue 2. C-terminal residues lacking detectable positions on the electron density map and therefore not involved in the structure below (20 C-terminal residues) are shown in italics. Individual sequences representing helices 1–6 are indicated with correspondingly colored lines. **b** Cartoon diagram illustrating the helical arrangement of the MA monomer with two possible orientations of the myristoyl group: sequestered (myr1A) and exposed (myr1B)

The asymmetric unit contains a dimer formed by two MA molecules. The structures of both MA subunits are virtually identical except for the position of the myristoyl group and the first five amino acids; the residual mean square deviation of Cα atoms for residues 7–92 is 0.306 Å (the N-terminal glycine is designated as residue 2). The monomeric subunit (Fig. 2) is a helical bundle comprising five alpha helices and a single-turn 3_10_-helix (helix 5). The CTRS region (spanning residues 45–62) is accessible on the surface of the molecule in an orientation similar to that observed for M-PMV MA [14, 32]. Despite the low sequence similarity of retroviral MA proteins (Table 2), which precluded the use of the molecular replacement approach in the structure determination, the position and orientation of the helices follow the structural pattern of other retroviral MA proteins.

**Table 2:** Comparison of myristoylated MMTV MA with other retroviral MAs

Genus	Species	PDB ID	Method	RMSD^a^	N^b^	%id^c^	%sim^c^
alpha	RSV	1A6S	NMR	4.68	72	13	19
beta	M-PMV	2F76	NMR	3.49	80	27	51
gamma	MoMuLV	1MN8	X-ray	4.67	80	15	31
delta	HTLV -2	1JVR	NMR	4.57	72	12	20
lenti	HIV-1	1HIW	X-ray	4.46	72	9	15

^a^Root mean square deviation of Cα-atoms

^b^Number of aligned residues for structural alignment

^c^Sequence identity and similarity, respectively, determined by global alignment using the Needle-Wunsch algorithm on the EMBL-EBI website with default parameters (http://www.ebi.ac.uk/Tools/psa/)

**Fig. 3:**
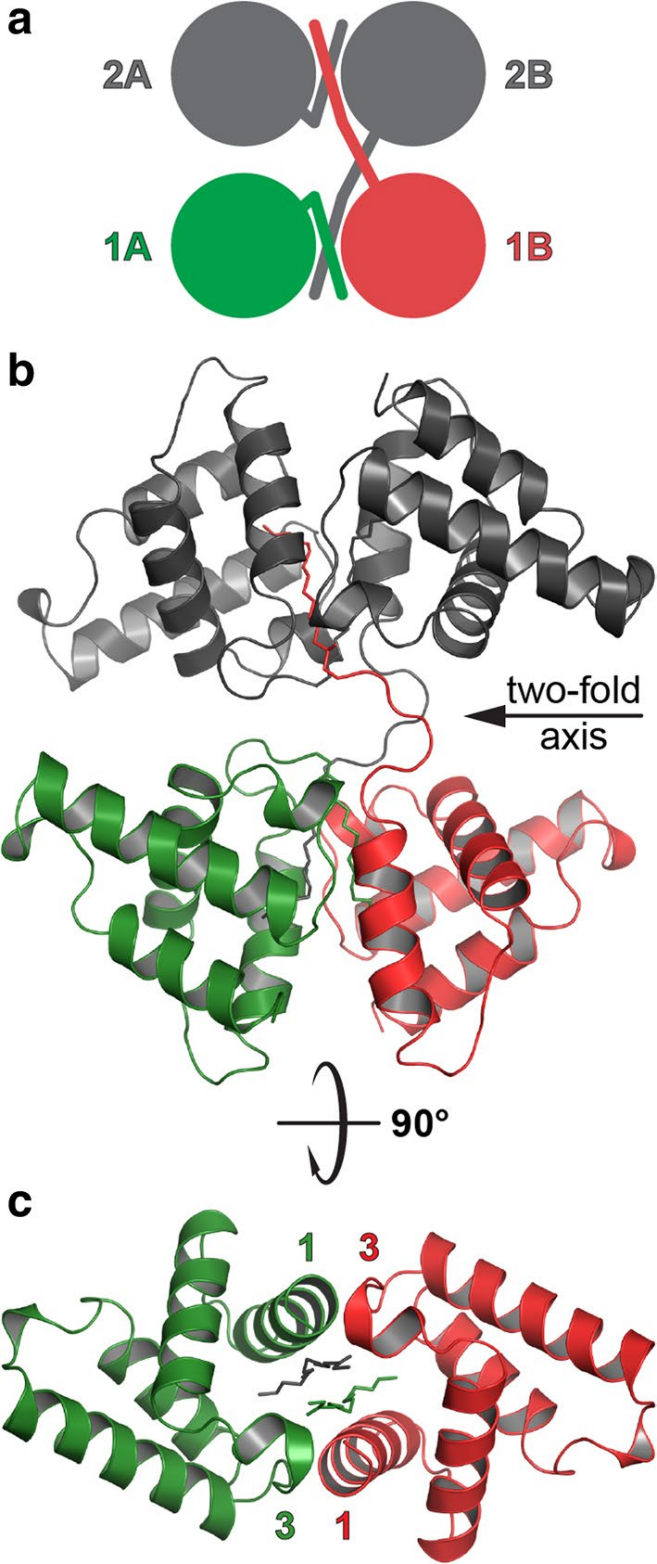
Organization of MMTV MA molecules in the crystal unit. **a** Schematic illustration of two dimer-forming MA molecules (*red* and *green*) that are connected with the neighboring dimer (*grey*) by swapping two myristoyl groups (*red* and *grey*). **b** Cartoon diagram illustrating the MA tetramer in an orientation perpendicular to the two-fold axis of symmetry. The *color* of individual myristoyl groups follows the same color scheme as in **a**. **c** Positions of both myristoyl groups in the hydrophobic pocket formed by the dimer interface are virtually identical regardless of the MA subunit to which they are covalently attached

Interestingly, the myristoyl group of one of the dimer-forming MA molecules is not buried in the hydrophobic core of the protein chain to which it is covalently attached, as is common for the sequestered form of myr(+) MA proteins [28, 30–32]. Instead, the myristoyl group is sequestered in the hydrophobic pocket formed by the interface of both MA molecules in the asymmetric dimer unit (Fig. 3). This interface is formed predominantly by helices 1 and 3. However, the myristoyl group of the second MA molecule in the dimer extrudes into the neighboring dimer unit. Therefore, the two nearest dimer units in the crystal are interconnected by two myristoyl groups, forming a tetramer of MA molecules around the two-fold axis of the *P3*_*1*_*21* space group (Fig. 3a, b). Regardless of the dimer unit to which the myristoyl groups are covalently attached, their positions in the hydrophobic interface are virtually symmetrical (Fig. 3c).

To analyze the relevance of the oligomeric organization in the protein crystal, we used PDBePISA [38], an interactive tool for the exploration of macromolecular interfaces. The PISA software detected several interfaces, of which the largest was 945 Å^2^ per MA monomer. The dominant interface lies between the two MA molecules in the single asymmetric unit. We did not identify any significant interface between MA dimers, with the exception of the interface represented solely by interconnecting myristoyl groups.

Because myr(+) MMTV MA predominantly forms dimers in solution, as confirmed by analytical ultracentrifugation analysis, the myristoyl swapping between the neighboring asymmetric units appears to be a specific result of crystal packing. In solution, the extruded myristoyl group is likely inserted into the hydrophobic pocket of its own dimer, forming a symmetric dimer with swapped myristoyl groups.

### Molecular dynamics simulation and calculation of interaction energies support the myristoyl swapping model in MMTV MA dimer formation

**Fig. 4:**
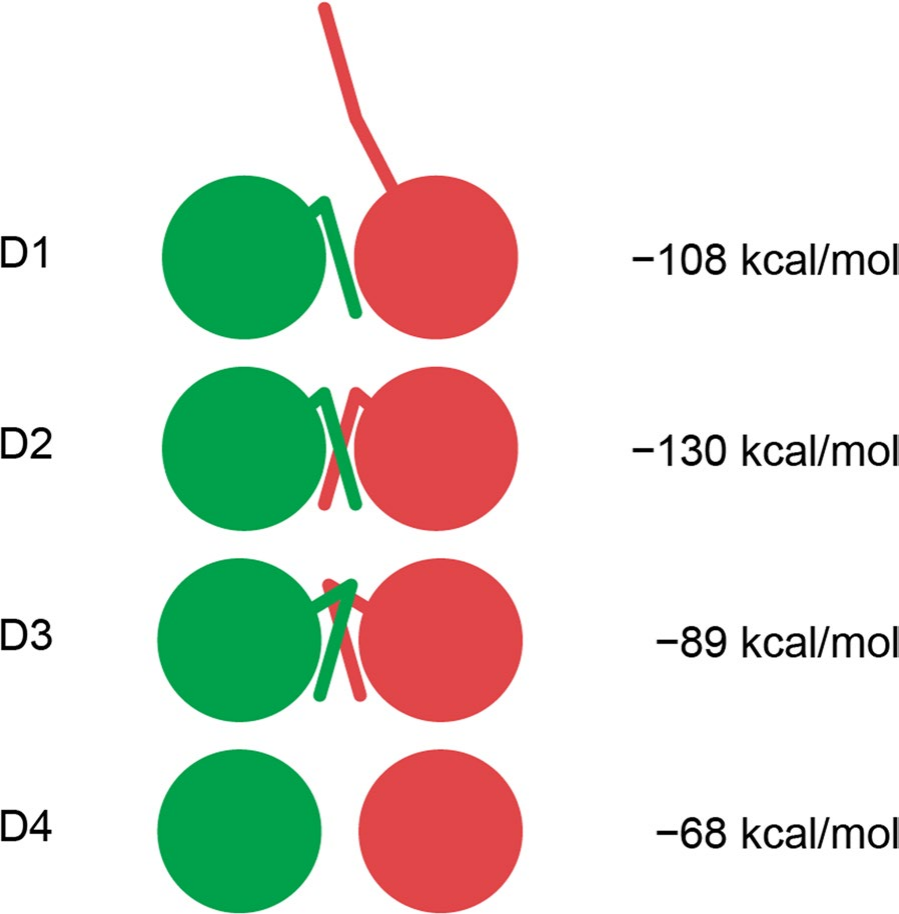
Schematic illustration of theoretical dimer formations adopted by the myristoylated MMTV MA and their calculated interaction energies. *D1* the asymmetric unit from the crystal structure, *D2* symmetric dimer with swapped myristoyls, *D3* symmetric dimer with unswapped myristoyls, *D4* symmetric dimer without myristoyl groups

Based on our experimental results, we propose that the swapping of myristoyl groups within the dimer is important for dimerization of MMTV MA. To support this proposal, we performed molecular dynamics (MD) simulations and calculated interaction energies for four theoretically possible MMTV MA dimers (Fig. 4). All the MD trajectories became stable after 6 ns of simulation. The root-mean-square deviations (RMSD) of the protein backbone fluctuated around 1 Å, while those of the myristoyl groups reached up to 1.7 Å in the case of dimer 3 (D3). Interaction energy calculations using the MM-PBSA approach showed the order of binding as follows: D2 (−130 kcal/mol, swapped myristoyls) >D1 (−108 kcal/mol, the asymmetric unit) >D3 (−89 kcal/mol, unswapped myristoyls) >D4 (−68 kcal/mol, nonmyristoylated). The results of the MD simulation indicate that myristoyl groups are energetically more favorable when swapped between the two dimer-forming MA molecules (Fig. 4, D2) than when unswapped (Fig. 4, D3). Furthermore, the absence of a single myristoyl group within the dimer interface causes the interaction energy to drop (Fig. 4, D1). To evaluate the relative contribution of individual residues to the dimerization, we performed a per-residue decomposition of the MM-PBSA interaction energies. The results, summarized in Table 3, show that the summed contribution of the myristoyl groups is three-fold higher for swapped myristoyl groups (−35.3 kcal/mol) than for unswapped myristoyl groups (−11.7 kcal/mol), which is comparable to the summed contribution of the most strongly contributing amino acid residues (−10 to −18 kcal/mol). These results support myristoyl swapping within the dimer as a driver for dimerization of MMTV MA.

**Table 3:** Contribution of individual residues to the dimerization of MMTV MA

D1			D2			D3			D4		
C^a^	Residue	E^b^	C	residue	E	C	Residue	E	C	Residue	E
A	Myr 1	−14.5	A	Myr 1	−18.0	A	Arg 18	−8.5	A	Arg 18	−9.6
B	Leu 11	−6.4	B	Myr 1	−17.3	B	Arg 18	−6.7	A	Leu 11	−4.9
A	Arg 23	−5.6	A	Arg 18	−9.0	B	Leu 11	−6.0	B	Leu 11	−4.8
B	Leu 52	−5.3	B	Arg 18	−9.0	B	Myr 1	−5.9	A	Lys 10	−4.7
A	Leu 52	−5.2	A	Leu 11	−5.7	A	Leu 11	−5.9	B	Lys 10	−4.5
B	Lys 10	−5.2	B	Leu 11	−5.7	A	Myr 1	−5.8	B	Leu 52	−4.5
A	Lys 10	−4.8	B	Lys 7	−5.1	A	Lys 10	−4.7	A	Leu 52	−4.5
B	Arg 23	−4.6	B	Leu 52	−4.8	B	Lys 10	−4.6	A	Lys 7	−2.3
A	Leu 11	−4.5	A	Lys 10	−4.7	A	Leu 52	−4.4	B	Glu 48	−2.1
B	Arg 18	−3.7	B	Lys 10	−4.6	B	Leu 52	−4.4	A	Asp 56	−2.1
B	Lys 7	−3.3	A	Leu 52	−4.6	A	Glu 48	−3.0	B	Lys 7	−2.0
B	Val 15	−3.0	A	Val 15	−3.0	A	Lys 7	−2.7	B	Val 15	−2.0
A	Lys 7	−2.9	B	Val 15	−3.0	B	Lys 7	−2.6	B	Asp 56	−2.0
A	Glu 22	−2.8	A	Lys 7	−2.5	B	Glu 48	−2.6	A	Val 15	−2.0
A	Arg 18	−2.6	A	Glu 48	−2.4	A	Val 15	−2.3	B	Glu 22	−1.8
B	Ser 14	−2.3	A	Gly 2	−2.2	B	Val 15	−2.2	B	Gly 8	−1.7
B	Glu 48	−2.0	B	Gly 8	−2.0	B	Gly 8	−1.9	B	Ser 14	−1.6
B	Leu 19	−1.9	B	Gly 2	−2.0	A	Gly 8	−1.7	A	Glu 48	−1.6
B	Gly 8	−1.8	B	Asp 56	−2.0	A	Glu 22	−1.6	A	Gly 8	−1.5
A	Val 15	−1.7	A	Gly 8	−1.9	A	Ser 14	−1.5	A	Ser 14	−1.4

First twenty residues with the lowest energy

^a^Chain

^b^Energy in kcal/mol

### Mutations within the dimerization interface impair MMTV MA dimerization

**Fig. 5:**
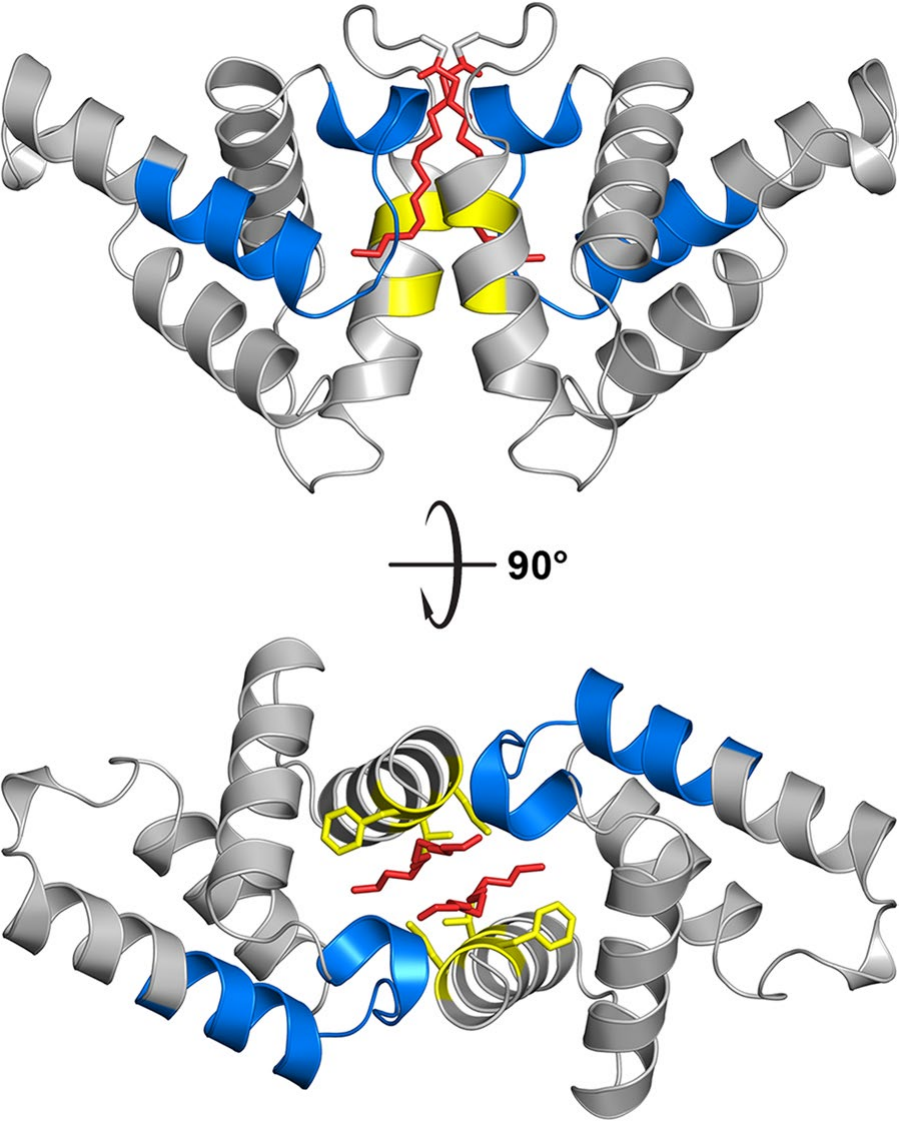
Cartoon diagram of myr(+) MA dimer showing positions of residues L11, F12, and V15. The residues are shown in *yellow*, myristoyl groups in *red*, and CTRSs in *blue*. Residues L11 and V15 are oriented towards the neighboring monomer whereas residue F12 is oriented towards the myristoyl group from the neighboring monomer. CTRS forms an important part of the dimerization interface

**Fig. 6:**
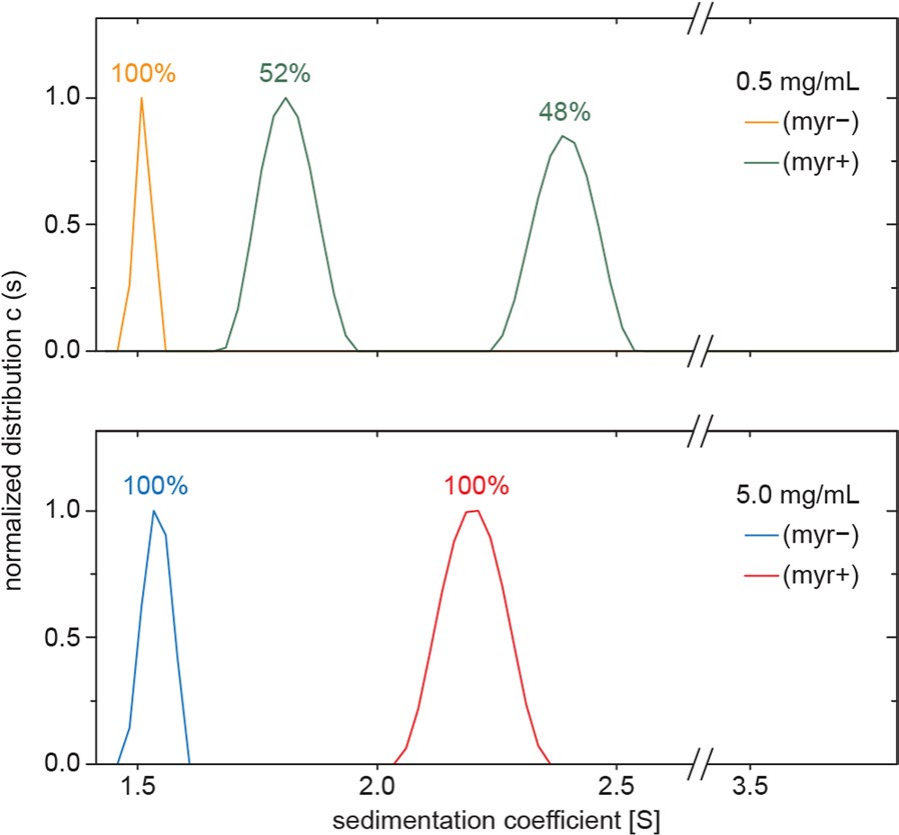
Dynamic equilibrium of the L11N/F12N/V15N triple mutant of myr(+) and myr(−) MA sedimenting species analyzed by analytical ultracentrifugation. Normalized continuous size distributions (c(s)) resulting from sedimentation velocity analyses were compared for myr(−) and myr(+) MAs at concentrations of 0.5 and 5.0 mg/mL. The percentages of monomeric, dimeric, and higher forms for individual MA forms in a single experiment are indicated above the peaks

To verify contribution of residues to MA dimerization, we mutated selected residues oriented to the interior of dimerization interface (Fig. 5). Because many residues from dimerization interface are located in the regions important for proper biological function of MMTV MA (myristoylation signal at the N-terminus, and cytoplasmic targeting/retention signal spanning residues 45–62 [15]), our choice of the residues available for mutation was restricted. We selected residues L11, F12, and V15 and prepared a triple mutant L11N/F12N/V15N (MA-3N) in both myristoylated and nonmyristoylated form. Analytical ultracentrifugation analyses of purified mutant myr(+) and myr(−) MA mutants were performed at two concentrations (0.5 and 5.0 mg/mL) under conditions identical to those used for WT MA. Figure 6 shows that mutations L11N, F12N, and V15N impaired MA dimerization. Contrary to the WT myr(−) MA, which partially formed dimers at the higher concentration, myr(−) MA-3N formed only monomers at both concentrations (s_20,w_ = 1.53 S). At the higher concentration, myr(+) MA-3N formed only dimers (s_20,w_ = 2.20 S) whereas wild-type myr(+) MA also formed a small amount of tetramers (2 %). At the lower concentration, myr(+) MA-3N formed monomers (s_20,w_ = 1.81 S; 52 %) and dimers (s_20,w_ = 2.39 S; 48 %) in contrast to wild-type myr(+) MA, which formed only dimers. The effect of L11N/F12N/V15N mutations on dimerization of myr(+) MA was lesser compared to the contribution of myristoyl group to this process. These results show that myristoylation itself is required, but not sufficient for efficient MMTV MA dimerization.

### Mutations affecting MMTV MA dimerization also influence extracellular particle production

To analyze impact of the mutations affecting MA dimerization on MMTV particle production, we performed pulse-chase experiments in transiently transfected human embryonic kidney 293T cells. For MMTV particle production, we used the chimeric proviral vector pSMt-HYB/D26A, which contains the heterologous M-PMV LTR promoter and a mutation inactivating the viral protease [11]. This construct facilitates detection of intracytoplasmic particles and it will be denoted hereafter as wild-type (WT).

**Fig. 7:**
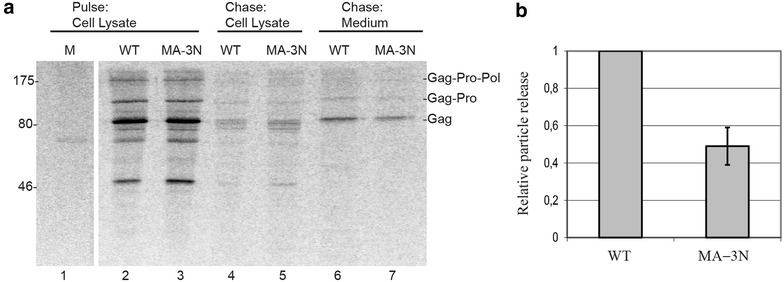
Mutations L11N, F12N and V15N in MA domain (MA-3N) reduce extracellular MMTV particle production. **a** Pulse-chase analysis of immature MMTV particles produced in transfected 293T cells. Cells expressed wild-type MMTV (WT) and the virus with the mutation in MA domain of Gag (MA-3N); *M* mock-transfected cells. Cells were labeled for 1 h with [^35^S]methionine and [^35^S]cysteine (*lanes 1*–*3*) and then chased for 12 h (*lanes* *4*–*5*). At both time points, cells were harvested, lysed, and subjected to immunoprecipitation. Released particles (*lanes* *6*–*7*) were collected from the culture media by ultracentrifugation through a 20 % sucrose cushion. Viral proteins were immunoprecipitated with polyclonal rabbit anti-CA serum, separated on a 10 % SDS-PAGE gel, and subjected to phosphorimager analysis. **b** Comparative analysis of released particle production. The efficiency was expressed as a share of the signal from the released particulate Gag from the total amount of Gag synthesized. Mutant MA-3N is shown relative to the WT (set to 1). Each *bar* represents the average of results from three independent experiments, and standard error of the mean is shown

Following transfection into 293T cells, WT and MA-3N mutant viruses were analyzed by metabolic labeling for their ability to express stable Gag polyproteins and produce extracellular immature particles (Fig. 7). Particle-producing cells were pulse-labeled for 1 h and chased for 12 h. Similar levels of stable Gag precursors were synthesized in all transfected cells and extracellular particles were observed for WT and mutant virus as well. As shown in Fig. 7a, the amount of pelletable structures in media produced by MA-3N mutant was lower than those produced by WT virus. To measure the relative efficiency of particle formation, the amount of extracellular assembled Gag as a fraction of total Gag expressed in cells was determined for each construct and then compared with that of WT (Fig. 7b). MA-3N virus produced particles with approximately half the efficiency of WT.

**Fig. 8:**
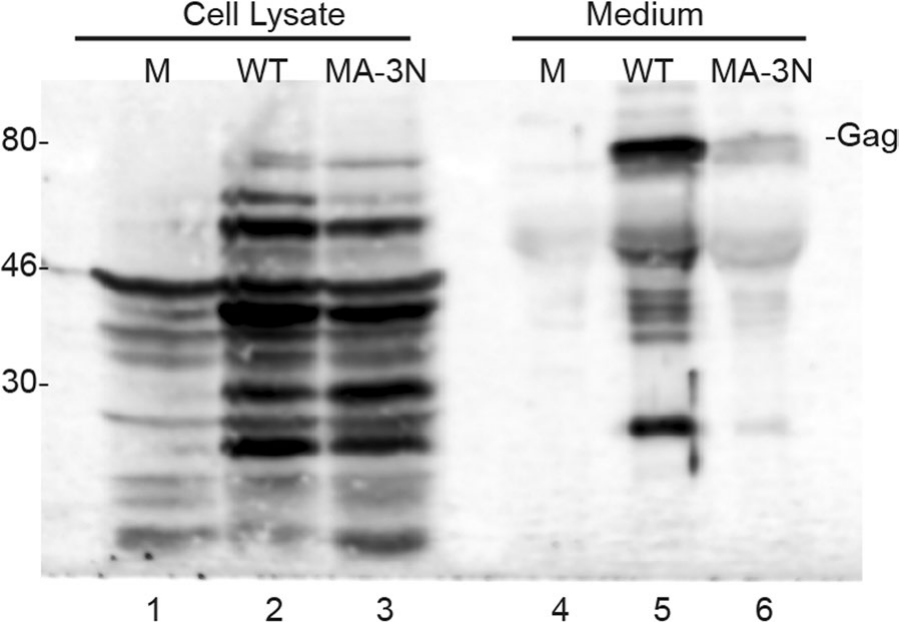
Western blot analysis of MMTV particles produced by rat mammary cells (RBA) transiently transfected with pSMt-HYB/D26A-derived constructs containing inactive viral protease. RBA cells expressed wild-type MMTV (WT: *lanes 2*, *5*) and virus with the mutated MA domain of Gag (MA-3N: *lanes* *3*, *6*). *Lanes 1* and *4* represent samples from mock-transfected cells. At 48 h post-transfection, cells were harvested and directly lysed in SDS protein loading buffer (Cell Lysate). The extracellular virus particles were isolated from culture media by ultracentrifugation through a 20 % sucrose cushion (Medium) and pellets were dissolved in SDS protein loading buffer. Proteins were separated on a 15 % SDS-PAGE gel and detected by Western blot assay using polyclonal anti-MMTV CA antiserum. The migration position of MMTV Gag is indicated

To verify the results obtained in human 293T cells, we used the rat mammary cell line RBA, which is suitable for production of MMTV upon transient transfection [39]. Because the expression level of MMTV proteins is significantly lower in RBA cells than in 293T cells, we examined the accumulation of assembled Gag in culture media by Western blot analysis after 48 h of production in transfected cells (Fig. 8). Proteins were separated on 15 % SDS PAGE gels and detected with a rabbit anti-MMTV capsid polyclonal antibody. Under these conditions, we were able to detect extracellular pelletable structures for both WT and MA mutant and as expected, the particle release of MMTV MA-3N from RBA cells was reduced. These results suggest that MMTV bearing mutations L11N, F12N and V15N in the MA domain exhibits reduced efficiency of extracellular particle production.

**Fig. 9:**
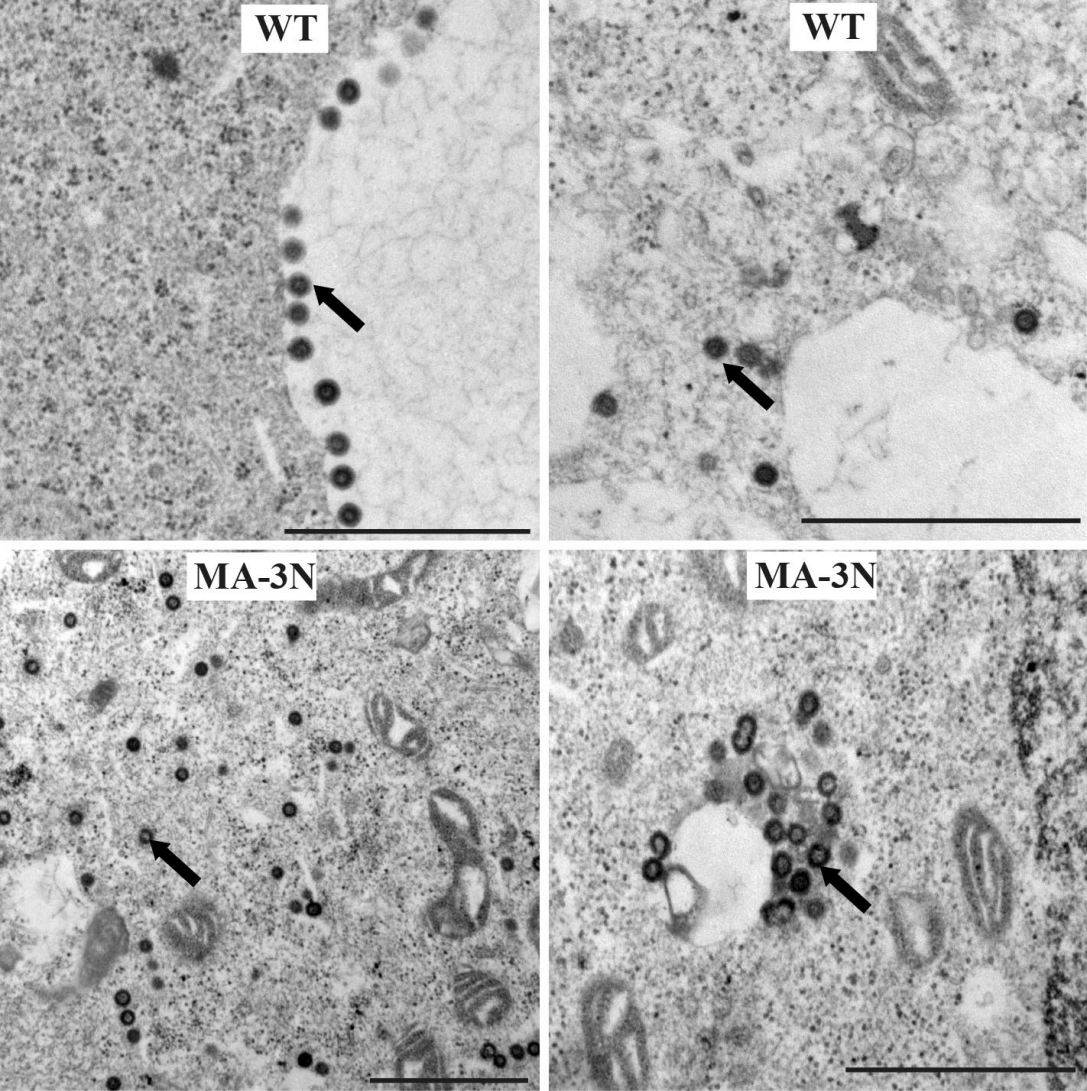
Thin-section EM analysis of immature intracytoplasmic MMTV particles formed by wild-type MMTV virus (WT) and the virus with the mutation in MA domain (MA-3N) in transfected 293T cells. *Black arrows* indicate immature viral particles. *Bars* correspond to 1 µm

To further investigate the effect of impaired MA dimerization on extracellular particle production, we analyzed transiently transfected 293T cells expressing WT and MA-3N virus by transmission electron microscopy (TEM) (Fig. 9). In contrast to the WT producing cells, only a minimum of particles budding from the plasma membranes and accumulation of both isolated and clusters of assembled particles were observed in cells expressing the MA-3N mutant virus. Aberrantly formed particles were detected with a low frequency similar for both WT and MA-3N MMTV. These observations suggest a reduced efficiency of the intracellular transport as an explanation for the partial defect of extracellular particle production in MA-3N mutant virus.

## Discussion

Here, we demonstrated that myristoylation stimulates dimerization of MMTV MA and identified structural requirements for this interaction. Molecular dynamics modeling based on our experimentally determined structure of myr(+) MA confirmed the key role of the myristoyl group for dimer formation and revealed energetically favorable binding of the myristoyl group within the dimer interface. Myristoylated MMTV MA shares structural similarity with other retroviral MAs, but it differs in the orientation of the sequestered myristoyl group. In other myr(+) MAs characterized to date, the myristoyl group is sequestered in the hydrophobic core of the molecule to which it is covalently attached [28, 30–32]. In contrast, the myristoyl group of MMTV MA is entirely buried in the dimerization interface between two MA molecules. Moreover, dimerization of MMTV MA supports the sequestered conformation of the myristoyl, which contrasts with the myristoyl switch model based on HIV-1 MA, in which oligomerization supports its extrusion [9].

Despite the fact that extrusion of one myristoyl group from one MMTV MA dimer unit to the neighboring asymmetric unit appears to be the result of crystal packing, it demonstrates an important aspect related to MA function. The process by which one MMTV MA molecule adopts an exposed myristoyl group conformation is analogous to the myristoyl switch model, in which the MA domain of a retroviral Gag polyprotein interacts with phospholipid membranes [30] and stimulates Gag multimerization. Thus, our results indicate that myr(+) MMTV MA can adopt both sequestered and exposed conformations analogous to those described for myr(+) HIV-1 MA.

The MMTV MA dimer interface forms a deep hydrophobic pocket that is energetically favorable for myristoyl binding. Our data from analytical ultracentrifugation clearly show that the myristoyl group stimulates MA dimer formation at high and low protein concentrations. The absence of the myristoyl group reduces the amount of MA dimer in solution even at high protein concentration (5 mg/mL). At a ten-fold lower concentration, myr(−) MA does not dimerize. It appears that the myristoyl group actively participates in formation of the dimer interface by interconnecting both monomeric MMTV MA units and that hydrophobic interactions are primarily responsible for dimer stability. Accordingly, no homotypic interactions were detected when myr(−) MMTV MA protein fused at the N-terminus with a reporter domain was tested for self-association in the yeast two-hybrid system [40]. Mutations of residues from dimerization interface (L11N/F12N/V15N), designed according to the MA crystal structure and molecular dynamic calculations, resulted in decreased MA dimerization. Interestingly, these mutations influenced also transport of assembled particles to PM and resulted in decreased production of extracellular particles. The current model describing the relocation of betaretroviral particles to PM involves anterograde transport mediated by microtubule-associated Env-containing vesicles [18, 41–43]. However, a detailed mechanism of this transport is not yet fully understood. These questions remain to be investigated.

**Fig. 10:**
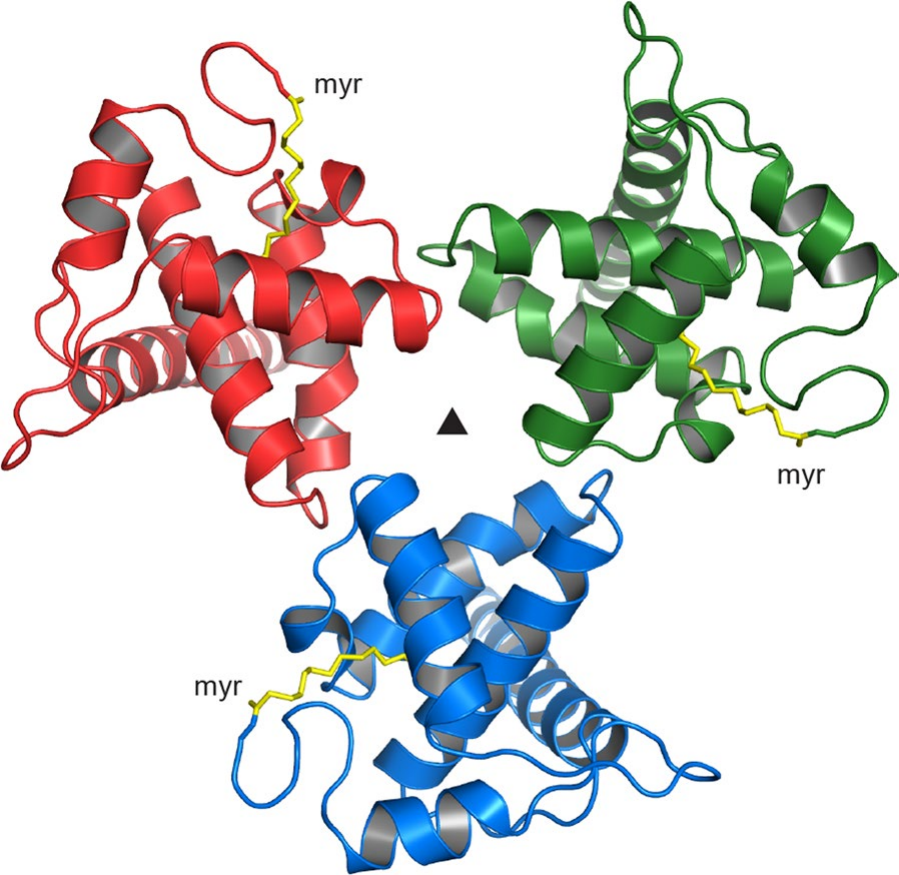
Position of the myristoyl groups within the HIV-1 MA trimer. The myristoyl groups (*yellow*) in the HIV-1 trimer do not participate in formation of the intermolecular interface. The model is based on the NMR structure of myr(+) HIV-1 MA (PDB ID 1UPH) superposed on the crystal structure of trimeric myr(−) HIV-1 MA (PDB ID 1HIW)

Myristoylation has been demonstrated to influence oligomerization of HIV-1 MA in solution; myr(−) MA is monomeric and myr(+) MA resides in monomer-trimer equilibrium [30]. Despite a known tendency of retroviral MAs to form dimers or trimers at high protein concentrations, they are predominantly monomeric in solution. To date, the only oligomeric state of a retroviral MA protein proven to be biologically relevant is the trimer initially discovered in the crystal structures of myr(−) simian immunodeficiency virus (SIV) MA [21] and myr(−) HIV-1 MA [33]. NMR spectroscopy showed that in solution, myr(+) HIV-1 MA also has a tendency to form trimers analogous to those observed in the crystal structures [30]. Although it was shown that myristoylation positively influences trimerization [30], the contribution of the myristoyl group to the trimerization of HIV-1 MA is of a different nature than our model proposes for dimerization of MMTV MA. In the HIV-1 trimer, the myristoyl groups are exposed and positioned outside of the trimer interface and thus cannot participate in its formation (Fig. 10).

In betaretrovirus assembly, there is no apparent requirement for MA interaction with the PM, and intracytoplasmic formation of immature particles instead relies on the internal scaffolding functions of domains located N-terminal to capsid protein [39, 40, 44–47]. In contrast to HIV-1 MA, the oligomerization capacity of betaretroviral M-PMV MA, which is capable of forming dimers and trimers in its myr(−) form [34, 48], is not stimulated by myristoylation [32]. Instead, the structural changes caused by the sequestered myristoyl negatively affect the oligomerization of the MA molecule. It was suggested that the oligomerization capacity of M-PMV MA is restored by conformational changes induced by the binding of MA to the membrane [32]. These differences from myristoyl-stimulated dimerization of MMTV MA suggest that betaretroviruses may have developed different strategies to sequester the MA myristoyl group until it needs to adopt an exposed conformation for membrane binding.

## Conclusions

We provide experimental data demonstrating that dimerization of MMTV MA is stimulated by its myristoylation. We clarified the molecular mechanism of this stimulation by determining the crystal structure of the myr(+) MMTV MA protein. Based on structural data and molecular modeling, we propose a model for symmetric dimer formation of myr(+) MMTV MA in solution. Our mechanism describing how dimerization of MMTV MA relies on swapped myristoyl groups represents a novelty not only among retroviral MA proteins but also for dimerization of any myristoylated protein. Our model suggests that oligomerization of betaretroviral MA protein is accompanied by sequestration of the myristoyl group that participates in the formation of the interaction interface. Although it contrasts with the model proposed for HIV-1 and other retroviruses that assemble with membrane support, our model is well-suited for intracytoplasmic betaretroviral assembly. Biological experiments further indicated that proper MA dimerization is important for efficient MMTV particle release.

## Methods

### DNA constructs

Plasmid pET22-MA-His used for the bacterial expression of MMTV MA protein was described previously [37]. Mutations of residues 11L, 12F and 15V to asparagines were introduced by PCR into pSMtv [11] plasmid containing MMTV gag gene sequence using forward mutagenic primer 5′-TGGGGGTCTCGGGCTCAAAAGGGCAGAAAAACAATGTTTCTAATTTACAAAGGCTCCTCTCAGAGAGGGGTC-3′ in combination with reverse primer 5′-TTTCAATGGCAGCGGTTCCC-3′ and pSMtv as template. Resulting PCR product was cleaved with AvaI–PflMI and ligated into pSMtv vector digested in the same way to produce pSMtv-MA-3N shuttle vector. Mutated gag sequence was used as a template for PCR using primers MA-Nde-F and MA-6H-Xho-R and the resulting mutant MA sequence was cleaved with NdeI–XhoI enzymes and ligated into identicaly prepared pET22b vector for the bacterial expression. The primer sequences used for this subcloning will be available upon request. In parallel, the complete gag sequence carrying the desired mutations was subcloned from pSmtv-MA-3N plasmid using KasI–XbaI enzymes and subcloned into identicaly treated MMTV proviral construct pSMt-HYB/D26A [11]. All DNA segments resulting from PCR were verified by sequencing.

### Expression and purification of recombinant MMTV MA protein

Recombinant myr(−) and myr(+) MMTV MA proteins were expressed and purified as previously described [37]. The molecular masses of the proteins were analyzed by mass spectrometry (MALDI TOF/TOF) as previously described [37]. To approximately determine the minimal detectable amount of myr(−) MA in the sample of myr(+) MA, we mixed a sample of myr(+) MA which we considered “100 %” myristoylated with a sample of myr(−) in the ratio 1000:1 (2:0.002 mg/mL) and analyzed the mixture by mass spectrometry (Additional file 1: Figure S1B). The 0.1 % concentration of myr(−) MA was detectable in the mixture and therefore we can state that the sample of myr(+) contained less than 0.1 % of myr(−) MA, which we suppose to not interfere with the subsequent analyses. After purification, the proteins were dialyzed against 50 mM Na_2_HPO_4_, 300 mM NaCl, 2.5 mM TCEP, pH 6.0 (buffer A), and concentrated to an appropriate concentration by ultrafiltration using an Amicon Ultra 3K device (Millipore).

### Analytical ultracentrifugation

Sedimentation velocity analyses were performed using a ProteomeLab XL-I analytical ultracentrifuge equipped with an An50Ti rotor (Beckman Coulter). Protein samples were concentrated to 0.5 or 5.0 mg/mL in buffer A, which was also used as a reference buffer. The experiments were carried out at 50,000 rpm and 4 °C; 200 absorbance scans were recorded at 6 min intervals with 30 µm spatial resolution at 280 nm for the diluted samples and at 250 nm for the concentrated samples with 12 and 3 mm Epon centerpieces, respectively. Buffer density and protein partial specific volume were estimated in SEDNTERP 1.09 (http://www.jphilo.mailway.com). Data were analyzed with SEDFIT 14.1 [49] using a c(s) continuous size distribution model. The approximate ratio of monomeric and dimeric forms was calculated from the peak areas.

### Protein crystallization

Myristoylated MMTV MA was concentrated to a final concentration of 10 mg/mL. Initial crystallization trials were performed with the help of a Crystal Gryphon crystallization workstation (Art Robbins Instruments) by sitting drop vapor diffusion method at 19 °C in 96-well plates; 0.2 μL protein solution was mixed with 0.2 μL reservoir solution and the mixture was equilibrated over 200 μL reservoir solution. The PEGs Suite and JSCG Core I Suite (QIAGEN) were used for the initial crystallization condition screen. Initial microcrystals appeared in several days under the following conditions: 0.2 M potassium chloride and 20 % PEG 3350. Further optimization involved changing to the hanging drop mode in 24-well crystallization plates (EasyXtal DG-Tool, QIAGEN). Final crystals were obtained by mixing 3 μL MMTV MA complex solution with 1 μL reservoir solution composed of 0.2 M potassium chloride and 20 % PEG 3350 as precipitant, and these were directly cryocooled in liquid nitrogen. For the phasing experiment, crystals were soaked for 10 min in a solution of 0.2 M potassium iodide and 20 % PEG 3350 and cryocooled in liquid nitrogen.

### Data collection and structure determination

Diffraction data for phasing were collected using a 1.5 Å wavelength for native and iodide-soaked crystals. Data were collected to 1.9 Å resolution at 100 K for both datasets. The native dataset for the final structure was collected using a 0.9184 Å wavelength at 100 K. All datasets were collected at the MX14.2 beamline at BESSY, Berlin, Germany [50], and processed using the XDS program [51]. The structure was solved with SHELXC/D/E programs [52] using HKL2MAP GUI [53] or GUI for SHELX programs [54]. Macromolecular phasing was performed according to the SIRAS method, using datasets from the native crystal and crystal soaked in potassium iodide (both measured using a 1.5 Å wavelength). The initial model was improved and rebuilt with the program Buccaneer [55]. This was followed by manual rebuilding with COOT [56] and refinement with Refmac5 [57] using the native dataset measured at 1.5 Å wavelength. At the end of this stage, the refinement statistics were as follows: R = 26.0 % (R_free_ = 32.4 %). The final model, calculated from the dataset measured at 0.9184 Å wavelength, was refined to R = 22.6 % (R_free_ = 26.3 %). The crystal parameters, data collection statistics, and refinement statistics are summarized in Table 1.

### Molecular dynamics simulation and calculation of interaction energies

Four possible dimers were derived from the crystal structure of myr(+) MMTV MA (Fig. 4). The first dimer (D1) was the asymmetric unit without any modifications. The second dimer (D2) was derived from the asymmetric unit by connecting the myristoyl group from monomer 2B to monomer 1B (Fig. 3a), which yielded a pseudo-symmetric dimer with swapped myristoyl groups. The third dimer (D3) was derived from D2 by swapping the connections of the proteins to the myristoyl groups, which yielded a dimer with unswapped myristoyl groups. The fourth dimer (D4) was derived from D2 by removing the myristoyl groups.

The systems were prepared for molecular dynamics (MD) simulations essentially as previously described [58], with a few modifications. The two histidine residues (both located on the protein surface far from the interface) were modeled in the Nε monoprotonated state. This setup was created in the LEaP module of AMBER14 [59]. The amino acid parameters were taken from the ff14SB AMBER force field [60], and the myristoyl parameters were obtained using RESP charge fitting [61] and GAFF parameters [62]. Chloride counterions were added to the solvated dimers to neutralize the total charge (four for D1–D3 and six for D4).

The initial relaxation included optimization of the myristoyl and Gly2 residues in 250 cycles of steepest descent. The other minimization steps, warming, and equilibration MD followed a previously described protocol [58]. The length of production MD was 10 ns. Monomer–monomer interaction energies were calculated using the MM-PBSA module of AMBER14 on 100 snapshots of the last 4 ns of the simulation. The Poisson-Boltzmann equation was solved, and Debye–Hückel screening with 150 mM ionic strength was used. The solvent-accessible surface-area-dependent term was employed for nonelectrostatic solvation free energies. The interaction energies were decomposed on a per-residue basis so that 1–4 interactions were added to either electrostatic or van der Waals contributions.

#### Cell lines and transfections

Human kidney embryonic cells 293T (ATCC) and rat mammary cells RBA (ATCC) were maintained in Dulbecco’s modified Eagle’s medium (DMEM) supplemented with 10 % fetal bovine serum and 20 mM l-glutamine (Sigma Aldrich). 293T cells were transfected by FuGENE HD (Roche Molecular Biochemicals) reagent and RBA cells were transfected using Lipofectamine LTX reagent (Invitrogen), both according to the manufacturer’s instructions.

#### Metabolic labeling and immunoprecipitation

Cells were grown on 60 mm culture dishes, 24 h post-transfection were 3 times washed with PBS buffer and then pulse-labeled for 60 min at 37 °C in 1 mL of DMEM without methionine and cysteine supplemented with 100 µCi of [^35^S]methionine-[^35^S]cysteine protein-labeling mix (MP Biochemicals). The label was chased for the desired period of time by replacing the labeling medium with complete DMEM. For analysis of total intracellular viral proteins, the cells were lysed in buffer containing 0.15 M NaCl, 50 mM Tris–HCl (pH 7.5), 1 % Triton X-100, and 1 % deoxycholate (Lysis Buffer). Cellular debris and nuclei were removed by centrifugation for 1 min at 14,000 rcf. The lysate was then adjusted to 0.1 % SDS and the cell-associated viral proteins were immunoprecipitated by rabbit polyclonal anti-CA antiserum for 1 h and after addition of protein A immobilized on Sepharose beads (Invitrogen) the suspension was incubated for 2 h. For analysis of released virus particles, tissue culture medium was first filtered through a 0.45 μm filter. Particles were then collected by centrifugation through a 20 % w/v sucrose cushion at 35,000 rpm for 1 h in a SW 41 Ti rotor (Beckman Coulter). Pelleted virions were dissolved in Lysis Buffer containing additional 0.1 % SDS and the virus-associated proteins were immunoprecipitated as described for cell lysates. Immunoprecipitates were examined by separation on 10 % SDS-PAGE gels followed by phosphorimager analysis. Band intensities for Gag were acquired on a Typhoon system using ImageQuant software (Amersham).

#### Western blotting

Transiently transfected RBA cells were grown on 100 mm culture dishes and harvested 48 h post-transfection. Trypsinized cells were pelleted for 1 min at 14,000 rcf and resuspended in 0.1 ml PBS and 0.2 ml of SDS protein loading buffer (PLB 2×) Virions from the culture supernatants were collected as described above for metabolically labeled proteins and resulting pellets were resuspended in 30 μl of PLB (2×). Analyzed proteins were separated on 10 % SDS-PAGE gel, blotted onto a nitrocellulose membrane, and detected by using rabbit polyclonal anti-CA antibody.

#### Electron microscopy

Examined cells were washed in PBS and fixed by 3 % glutaraldehyde in 0.1 M cacodylate buffer, pH 7.4. The cells were postfixed with 1 % osmium tetroxide, dehydrated in graded ethanol solutions and embedded in epoxy resin AGAR 100. Ultrathin sections (70 nm) were stained with uranyl acetate and lead citrate. The samples were analyzed with a JEOL JEM-1200EX electron microscope operated at 60 kV.

### Availability of supporting data

The structure of myristoylated MMTV MA protein was deposited in the Protein Data Bank under PDB ID 4ZV5.

## Additional file

10.1186/s12977-015-0235-8 MS MALDI TOF/TOF analysis of myristoylated, myr(+), and nonmyristoylated, myr(−), MMTV MA. (A) The sample of myristoylated MA contained myr(+) MA (m/z = 13006). The sample of nonmyristoylated MA contained myr(−) MA (m/z = 12795) and MA with uncleaved initial methionine (m/z = 12927). (B) To approximately determine the minimal detectable amount of myr(−) MA in the sample of myr(+) MA, the sample of myr(+) MA was mixed with the sample of myr(−) in the ratio 1000:1 (2:0.002 mg/mL). This amount of myr(−) MA was detectable whereas the sample of myr(+) contained no detectable amount of myr(−) MA.

## Abbreviations

CTRS: cytoplasmic targeting/retention signal
HIV-1: human immunodeficiency virus type 1
MA: matrix
MMTV: mouse mammary tumor virus
M-PMV: Mason-Pfizer monkey virus
myr(+): myristoylated
myr(–): nonmyristoylated
PM: plasma membrane
