# Supplementary material for: Myristoylation drives dimerization of matrix protein from mouse mammary tumor virus
Source: Retrovirology. 2016 Jan 5;13:2. doi: 10.1186/s12977-015-0235-8 (PMC4700671; doi:10.1186/s12977-015-0235-8)
Supplement: Supplementary file 1 — 10.1186/s12977-015-0235-8 MS MALDI TOF/TOF analysis of myristoylated, myr(+), and nonmyristoylated, myr(−), MMTV MA. (A) The sample of myristoylated MA contained myr(+) MA (m/z = 13006). The sample of nonmyristoylated MA contained myr(−) MA (m/z = 12795) and MA with uncleaved initial methionine (m/z = 12927). (B) To approximately determine the minimal detectable amount of myr(−) MA in the sample of myr(+) MA, the sample of myr(+) MA was mixed with the sample of myr(−) in the ratio 1000:1 (2:0.002 mg/mL). This amount of myr(−) MA was detectable whereas the sample of myr(+) contained no detectable amount of myr(−) MA. [file 12977_2015_235_MOESM1_ESM.pdf]

Myristoylation drives dimerization of matrix protein from mouse mammary tumor virus

Michal Doležal[†1], Aleš Zábranský[†1], Jiří Dostál[1], Ondřej Vaněk[2], Jiří Brynda[1], Martin Lepšík[1], Romana Hadravová[1] and Iva Pichová[1,*]

[1]Institute of Organic Chemistry and Biochemistry, Academy of Sciences of the Czech Republic, v.v.i., Flemingovo nám. 2, 166 10 Prague, Czech Republic

[2]Department of Biochemistry, Faculty of Science, Charles University in Prague, Hlavova 8, 128 40 Prague, Czech Republic

[†]Contributed equally

*Corresponding author

mailing address: IOCB, Flemingovo nám. 2, 166 10 Prague 6, Czech Republic

e-mail: iva.pichova@uochb.cas.cz

phone number: +420220183251

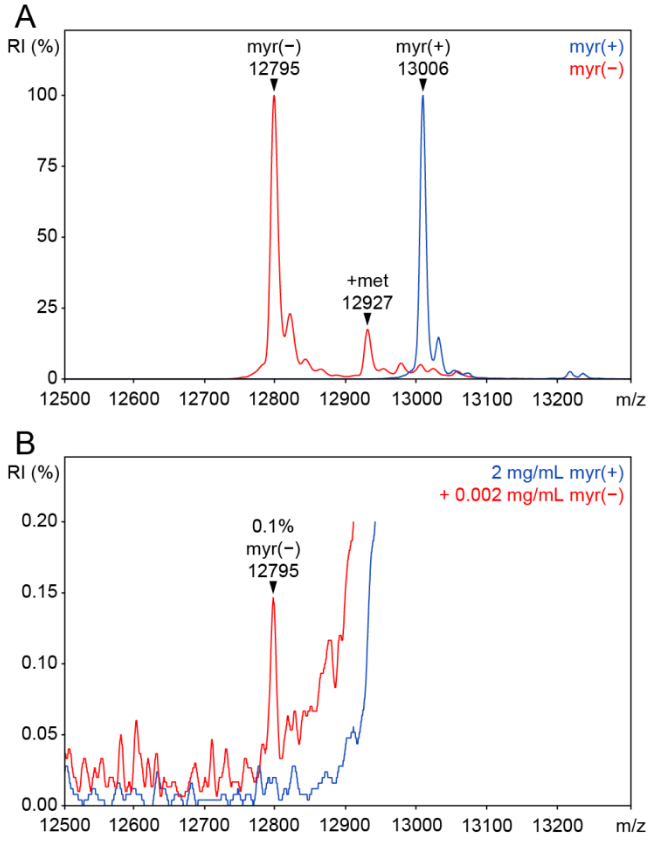

Suplementary Figure 1

MS MALDI TOF/TOF analysis of myristoylated, myr(+), and nonmyristoylated, myr(−), MMTV MA. (A) The sample of myristoylated MA contained myr(+) MA (m/z = 13006). The sample of nonmyristoylated MA contained myr(−) MA (m/z = 12795) and MA with uncleaved initial methionine (m/z = 12927). (B) To approximately determine the minimal detectable amount of myr(−) MA in the sample of myr(+) MA, the sample of myr(+) MA was mixed with the sample of myr(−) in the ratio 1000:1 (2:0.002 mg/mL). This amount of myr(−) MA was detectable whereas the sample of myr(+) contained no detectable amount of myr(−) MA.
